# Real-World Evidence Demonstrating Nemolizumab's Rapid Efficacy in Recalcitrant Uremic Pruritus

**DOI:** 10.7759/cureus.91929

**Published:** 2025-09-09

**Authors:** Hunter Dalisay, Harshitha Segabandi, Kostandin Valle, Daniel Tinker

**Affiliations:** 1 Dermatology, Dermatological Care, St. Louis, USA; 2 Dermatology, University of Missouri, Columbia, USA

**Keywords:** chronic kidney disease, hemodialysis, il-31, interleukin-31, nemolizumab, pruritus treatment, recalcitrant uremic pruritus, uremic pruritus

## Abstract

Uremic pruritus is a common and distressing condition in patients with advanced chronic kidney disease (CKD). It often leads to disruption of sleep habits, impaired mental health, and reduced quality of life. Nemolizumab is an interleukin-31 receptor subunit alpha (IL-31R𝛼) antagonist that is currently approved to treat prurigo nodularis (PN) and atopic dermatitis (AD). However, its effectiveness in treating uremic pruritus remains uncertain, as it has only been explored in a limited number of small studies. We present a case of a 69-year-old man on long-term hemodialysis who presented with refractory uremic pruritus. The patient initially reported a pruritus numerical rating scale (NRS) score of 8 out of 10, indicating severe itch on a scale where 0 represents no itch and 10 represents the worst itch imaginable. Despite adequate six-week trials of triamcinolone acetonide 0.1% ointment twice daily, narrowband ultraviolet B (NB-UVB) phototherapy three times weekly, N-acetylcysteine (600 mg twice daily), and gabapentin (300 mg three times daily), his debilitating itch persisted. Consequently, treatment with nemolizumab was initiated. Just two weeks after the initial dose, the patient reported an NRS score of 0, indicating complete relief from pruritus, along with a reduction in excoriated lesions observed on physical examination. The patient reported no side effects from nemolizumab. This case highlights the potential of nemolizumab as an effective and safe treatment option for recalcitrant uremic pruritus. Further exploration of its therapeutic applications in this condition and other vexing-to-treat pruritic conditions is warranted.

## Introduction

Chronic pruritus is a commonly reported and difficult-to-treat symptom in patients with end-stage kidney disease (ESKD), especially those receiving hemodialysis, in whom the condition is often referred to as uremic pruritus. The pathophysiology of uremic pruritus is not fully understood, but complex interactions involving pruritoceptive, neuropathic, neurogenic, and psychogenic factors may contribute [1]. In hemodialysis patients, adequate clearance of pruritogenic compounds is a predictor of pruritus intensity, suggesting that deposition of these by-products in the skin contributes to itch [1]. Furthermore, the development of pruritus involves various inflammatory pathways. These include the release of histamine and interleukins, as well as activation of numerous receptors, including opioid receptors, C-fibers, and IL-31R𝛼 [2,3]. IL-31, a cytokine secreted by type 2 helper-T cells (Th2 cells), is strongly associated with pruritus, with elevated serum levels observed in patients undergoing hemodialysis [4]. Given the inflammatory pathways involved in this condition, first-line management focuses on emollients and moisturizers, while additional treatment options include topical steroids, phototherapy, and immunosuppressant medications [3]. These therapies are commonly supplemented by moisturizers for maximum symptom relief [3]. For recalcitrant cases, treatment can be difficult, and symptoms can significantly impact sleep, mental well-being, and overall quality of life [3]. 

Nemolizumab is a humanized monoclonal antibody that binds to IL-31R𝛼. It has been approved for the treatment of atopic dermatitis (AD) and prurigo nodularis (PN) [2,5]. A randomized clinical trial demonstrated that in patients with PN, nemolizumab significantly improved itch intensity and required less rescue therapy compared to the placebo [6]. The most common adverse effects during this trial of nemolizumab therapy included AD and headache [6]. The patients in this clinical trial who developed AD experienced minor symptoms, which were well managed with topical treatments, and continued nemolizumab [6]. Despite its proven efficacy in randomized clinical trials to relieve itch, few studies have been completed examining its use in uremic pruritus. A phase II randomized clinical trial in Japan compared nemolizumab at various concentrations with placebo and nalfurafine hydrochlorine, using the pruritus visual analog scale (VAS) as the primary endpoint [7]. While no statistically significant differences were found between nemolizumab and placebo, nemolizumab groups showed greater mean VAS reductions, particularly at the 0.5 mg/kg dose, without clinically significant adverse effects [7]. The study’s interpretation was limited by a small sample size of 48 patients and the low sensitivity of the VAS [7]. Nevertheless, the use of nemolizumab in uremic pruritus remains investigational. Herein, we present the case of a 69-year-old male with ESKD on hemodialysis and treatment-refractory uremic pruritus who experienced a rapid and near-complete resolution of symptoms following treatment with nemolizumab.

## Case presentation

A 69-year-old man with a history of ESKD on dialysis presented to the clinic with a chief concern of dry, itchy, and scaly skin on the lower trunk for several months. Physical examination revealed widespread hemorrhagic, excoriated patches and plaques distributed on the trunk and extremities. His initial treatment regimen included triamcinolone acetonide 0.1% topical ointment twice daily, narrowband ultraviolet B (NB-UVB) phototherapy three times weekly, and gabapentin 300 mg three times daily. Additionally, oral N-acetylcysteine, oral antihistamines, and over-the-counter anti-itch lotions were utilized for symptom management. At his follow-up appointment, the patient reported continued pruritus. Physical examination revealed erythematous nodules with central ulceration and progression of excoriated chest and back lesions. Despite minimal improvement, his pruritus remained severe, scoring 8 out of 10 on the numerical rating scale for itch (NRS).

Given the failed medical interventions and that the patient was not a candidate for immunosuppressant medications, nemolizumab was discussed. Our patient was concomitantly taking medications with narrow therapeutic indices, including theophylline and gabapentin. This precluded the initiation of more traditional first-line treatments for uremic pruritus, underscoring the management challenge physicians face when caring for patients with this condition. 

The prescribing information was carefully reviewed and nemolizumab did not have any interactions with theophylline or any other medications our patient was concomitantly taking. The patient consented to treatment and received an initial injection of nemolizumab 30 mg subcutaneously. It is important to note that current guidelines for nemolizumab therapy support a loading dose of 60 mg [8]. Despite a reduced loading dose, at his follow-up visit two weeks later, the patient noted significant improvement with almost complete resolution of pruritus, as indicated by an NRS itch severity score of 0. Due to his profound positive response, a second 30 mg injection of nemolizumab was administered subcutaneously to the periumbilical region at week four. Eight weeks after initial injection, the patient again reported an NRS itch severity score of 0, and a third 30 mg injection of nemolizumab was administered subcutaneously to the periumbilical region, with a plan to continue the injections once every four weeks. Eight months later, the patient's uremic pruritus has remained well-controlled on nemolizumab and emollients, with no adjustments to his theophylline dosage.

## Discussion

Pruritus involves a multifactorial mechanism characterized by the retention of uremic toxins, elevated inflammatory pathways, a disrupted immune system, and loss of skin barrier functionality [9]. Clinically, uremic pruritus often presents as an intense itch affecting the trunk and extremities with associated excoriations. Patients receiving hemodialysis therapy typically have elevated levels of IL-31; consequently, uremic pruritus, like other pruritic diseases, is associated with a signaling cascade involving IL-31 [10]. In such patients, the itch sensation and associated scratching behavior drive the pathological process referred to as the “itch-scratch” cycle [11]. When the skin and epithelial cells are damaged from scratching, inflammatory cytokines, such as IL-31, are released that promote itch signaling to the brain, thereby perpetuating the itch-scratch cycle [11]. IL-31 binds to a heterodimeric receptor composed of IL-31R𝛼 and the oncostatin M receptor (OSMR) on keratinocytes, epithelial cells, and unmyelinated C-fibers [12]. Binding to these receptors leads to physiologic activation of the JAK/STAT pathway, particularly STAT3, which mediates nerve signaling involved in the itch-scratch cycle [12]. Additionally, IL-31 activation causes phosphorylation of extracellular signal-regulated kinases 1 and 2 (ERK1/2) in neurons, which further potentiates nerve signaling associated with itch [13-15]. By antagonizing the receptors involved in this signaling cascade, nemolizumab has the potential to relieve the psychogenic sensation of itch associated with uremic pruritus while also downregulating inflammatory pathways.

The psychosocial impact that pruritus has on this patient population is immense. Because of impaired renal clearance and myriad concomitant medications, the risk of drug-drug interactions obfuscates effective treatment options for this condition. In our case, the patient was taking both theophylline and gabapentin, medications known for their narrow therapeutic windows, yet nemolizumab was initiated safely without any observed interactions. As a monoclonal antibody, nemolizumab is not affected by reduced renal or hepatic function and does not interact with other drugs cleared through these pathways, a finding consistent with results from the pivotal phase III clinical trials [8]. With limited prior research on this condition, effective treatment options for uremic pruritus remain sparse, reinforcing the need for further exploration of the therapeutic utility of nemolizumab. This case report highlights the therapeutic potential of IL-31 blockade in uremic pruritus.

## Conclusions

Current treatment options for uremic pruritus remain limited in efficacy, underscoring the need for novel therapeutic strategies. Although the exact pathogenesis of uremic pruritus remains unclear, several hypotheses have been proposed, including direct local tissue damage from uremic byproducts and dysregulated nerve signaling. IL-31, a cytokine hypothesized to play a role in dysregulated neuronal signaling, is inhibited by the recently approved monoclonal antibody nemolizumab. Herein, the authors present a 69-year-old male with ESKD and recalcitrant uremic pruritus on myriad prescription medications who experienced a rapid, deep, and sustained clinical response to treatment with nemolizumab. A quantifiable measure of response was a decrease in NRS itch severity score from 8 to 0 after just one 30 mg dose of nemolizumab. Eight months later, he continues to tolerate the nemolizumab treatment with 30 mg once every four weeks and remains disease-free. The patient’s significant clinical improvement suggests nemolizumab’s potential as an efficacious therapeutic option for patients with recalcitrant uremic pruritus, without the risk of drug-drug interactions that other next-best interventions are known to provoke.
